# Supplementary material for: Cell Cycle Constraints and Environmental Control of Local DNA Hypomethylation in α-Proteobacteria
Source: PLoS Genet. 2016 Dec 20;12(12):e1006499. doi: 10.1371/journal.pgen.1006499 (PMC5172544; doi:10.1371/journal.pgen.1006499)
Supplement: S1 Text — Table A. Strains and plasmids used in this study. Table B. Oligonucleotides used in this study. (DOCX) [file pgen.1006499.s001.docx]

**Supporting Information**

**Strains and plasmids construction**

For constitutive expression of *ccrM*, Pl*_ac_*-*ccrM* was transduced from LS1 into the NA1000 *WT* strain by φCr30-mediated transduction and selection on PYE kanamycin plates.

To create *lacZ* transcriptional fusions, promoter regions were cloned into pRKlac290 as follows:

pSA488: a 597-bp DNA fragment was amplified by PCR with primers P169_E/P169_X, digested with *Eco*RI and *Xba*I, and ligated into pRKlac290, cut with the same enzymes.

pSA389: the mutated P*169* was amplified by PCR with primers P169_E/P169_X from a synthetic fragment (sequence appended below), digested with *Eco*RI and *Xba*I, and ligated into pRKlac290, cut with the same enzymes.

pSA500: a 553-bp DNA fragment was amplified by PCR with primers P1149_E/P1149_X, digested with *Eco*RI and *Xba*I, and ligated into pRKlac290, cut with the same enzymes.

pSA499: a 591-bp DNA fragment was amplified by PCR with primers P2901_E/P2901_H, digested with *Eco*RI and *Hind*III, and ligated into pRKlac290, cut with the same enzymes.

pSA390: the mutated P*2901* was amplified by PCR with primers P2901_E/P2901_H from a synthetic fragment (sequence appended below), digested with *Eco*RI and *Hind*III, and ligated into pRKlac290, cut with the same enzymes.

pSA296: a 591-bp DNA fragment was amplified by PCR with primers P2902_AS_E/P2902_AS_H, digested with *Eco*RI and *Hind*III, and ligated into pRKlac290, cut with the same enzymes.

pSA313: a 619-bp DNA fragment was amplified by PCR with primers P3247_AS_E/P3247_AS_X, digested with *Eco*RI and *Xba*I, and ligated into pRKlac290, cut with the same enzymes.

pSA527: the construct carrying the attenuated *E. coli* T5 promoter followed by three GANTCs overlapping two CtrA boxes was created by digesting a 153-bp synthetic fragment (sequence appended below) with *Eco*RI and *Xba*I and ligating into pRKlac290, cut with the same enzymes.

pSA259: a 530-bp DNA fragment was amplified by PCR with primers P_SMa1635_E/P_SMa1635_X from *S. meliloti* genomic DNA, digested with *Eco*RI and *Xba*I, and ligated into pRKlac290, cut with the same enzymes.

pSA260: a 635-bp DNA fragment was amplified by PCR with primers P_SMa2245_E/P_SMa2245_X from *S. meliloti* genomic DNA, digested with *Eco*RI and *Xba*I, and ligated into pRKlac290, cut with the same enzymes.

Plasmids for constitutive expression (from P*_van_* or P*_lac_*) were constructed as follows:

pSRK-*ccrM^Cc^*: *ccrM* ORF was amplified by PCR with primers ccrM_N (with *Nde*I site overlapping the start codon) and ccrM_X (with *Xba*I site flanking the stop codon) and cloned into pSRKGm, restricted with *Nde*I and *Eco*RI.

pMT335-*ccrM^Cc^*: *ccrM* ORF was amplified by PCR with primers ccrM_E/ccrM_X, digested with *Eco*RI and *Xba*I, and ligated into pMT335, restricted with the same enzymes.

pMT335-*TA*: the construct to express the methylase from *T. acidophilum* in *C. crescentus* was made by ligating a synthetic fragment (sequence appended below) into pMT335, using *Nde*I and *Eco*RI.

pMT335-*HP*: the construct to express the methylase from *H. pylori* in *C. crescentus* was made by ligating a synthetic fragment (sequence appended below) into pMT335, using *Nde*I and *Eco*RI.

pMT335-*hinf*: the construct to express the methylase from *H. influenzae* in *C. crescentus* was made by ligating a synthetic fragment (sequence appended below) into pMT335, using *Nde*I and *Eco*RI.

pMT335-*mucR^Sm^*: *SMc00058* ORF was amplified by PCR with primers Sm_mucR_N (with *Nde*I site overlapping the start codon) and Sm_mucR_E (with *Eco*RI site flanking the stop codon) from *S. meliloti* genomic DNA and cloned into pMT335, restricted with *Nde*I and *Eco*RI.

**Table A. Strains and plasmids used in this study**

| ***Caulobacter crescentus*** | **Relevant characteristics** | **Reference or source** |
| --- | --- | --- |
| NA1000 | syn-1000, synchronizable variant of strain CB15 | [1] |
| Δ*mucR1*Δ*mucR2* | NA1000 derivative with in-frame deletion of *mucR1* and *mucR2* | [2] |
| LS1 | NA1000 *bla6* *rsaA2* with P*_lac_*-*ccrM* integrated at the *ccrM* locus | [3] |
| NA1000 *ccrM*::P*_lac_*-*ccrM* | NA1000 derivative with P*_lac_*-*ccrM* integrated at the *ccrM* locus, Kana^R^ | This work |
| Δ*mucR1*Δ*mucR2*  *ccrM*::P*_lac_*-*ccrM* | Δ*mucR1*Δ*mucR2* derivative with P*_lac_*-*ccrM* integrated at the *ccrM* locus, Kana^R^ | This work |
| *lon*Ω | NA1000 derivative with an Ω cassette inserted in the *lon* gene, Spec^R^ | [4] |
| Δ*phoB::*Ω | NA1000 derivative with an Ω cassette inserted in the *phoB* gene, Spec^R^ | [5] |
| ***Sinorhizobium meliloti*** |  |  |
| Rm2011 | Wild-type strain | [6] |
| Rm101 | Rm2011 with Spc^R^ cassette inserted into the *Pma*CI site of *mucR* | [7] |
| ***Escherichia coli*** |  |  |
| EC100D | Cloning strain | Epicentre Technologies |
| S17-1 | For plasmid mobilization | [8] |
| **Plasmid** | **Relevant characteristics** | **Reference or source** |
| pRKlac290 | *lacZ* transcriptional fusion vector, pRK290 derivative; Tet^R^ | [9] |
| pSA205 | pRKlac290 derivative carrying P*_hvyA_-lacZ*; Tet^R^ | [10] |
| pJS70 | pRKlac290 derivative carrying P*_pilA_-lacZ*; Tet^R^ | [11] |
| pSA488 | pRKlac290 derivative carrying P*169-lacZ*; Tet^R^ | This work |
| pSA389 | pRKlac290 derivative carrying P*169*-lacZ*; Tet^R^ | This work |
| pSA500 | pRKlac290 derivative carrying P*1149-lacZ*; Tet^R^ | This work |
| pSA499 | pRKlac290 derivative carrying P*2901-lacZ*; Tet^R^ | This work |
| pSA390 | pRKlac290 derivative carrying P*2901*-lacZ*; Tet^R^ | This work |
| pSA296 | pRKlac290 derivative carrying P*2902_AS-lacZ*; Tet^R^ | This work |
| pSA313 | pRKlac290 derivative carrying P*3247_AS-lacZ*; Tet^R^ | This work |
| pSA527 | pRKlac290 derivative carrying an attenuated phage T5 promoter followed by two CtrA-boxes; Tet^R^ | This work |
| pSA259 | pRKlac290 derivative carrying P*_SMa1635_-lacZ*; Tet^R^ | This work |
| pSA260 | pRKlac290 derivative carrying P*_SMa2245_-lacZ*; Tet^R^ | This work |
| pSRK | pBBR1MCS-5 derived broad host range vector containing *lac* promoter, *lacI*^q^, l*acZ*α^+^; Gm^R^ | [12] |
| pSRK-*ccrM^Cc^* | pSRKGm derivative carrying the *C. crescentus ccrM* ORF; Gm^R^ | This work |
| pMT335 | High copy number plasmid for inducible expression; P*_van_*, Gm^R^ | [13] |
| pMT335-*ccrM^Cc^* | pMT335 derivative carrying the *C. crescentus ccrM* ORF; Gm^R^ | This work |
| pMT335-*mucR1* | pMT335 derivative carrying the *C. crescentus mucR1* ORF; Gm^R^ | [2] |
| pMT335- *mucR1-long* | pMT335 derivative carrying the *C. crescentus mucR1* with an N-terminal extension; Gm^R^ | [2] |
| pMT335-*TA* | pMT335 derivative carrying the methylase from *Thermoplasma acidophilum*; Gm^R^ | This work |
| pMT335-*HP* | pMT335 derivative carrying the methylase from *Helicobacter pylori*; Gm^R^ | This work |
| pMT335-*hinf* | pMT335 derivative carrying the methylase from *Haemophilus influenzae*; Gm^R^ | This work |
| pMT335-*mucR^Sm^* | pMT335 derivative carrying the *S. meliloti mucR* ORF; Gm^R^ | This work |

**Table B. Oligonucleotides used in this study**

| **Primer** | **Sequence 5’-3’ (Restriction sites underlined)** |
| --- | --- |
| P169_E | AAA AAA GAA TTC GCT TGC CAG CAT TGT TCG CAA A |
| P169_X | AAA AAA TCT AGA ATT AGG TTT CTA TAC AGC TTC A |
| P1149_E | AAA GAA TTC ACC GAC ATC GGC CTC GTG TGG AA |
| P1149_X | AAA TCT AGA GTC AGC AGC GAA GCT GGG TGT GAA |
| P2901_E | AAA AAA GAA TTC AAC CAT TAT AAT TCG GGC ACA AA |
| P2901_H | AAA AAA AAG CTT GTA GCC GGT CAG GCG CTG GAA |
| P2902_AS_E | AAA AAA GAA TTC GTA GCC GGT CAG GCG CTG GAA |
| P2902_AS_H | AAA AAA AAG CTT AAC CAT TAT AAT TCG GGC ACA AA |
| P3247_AS_E | AAA AAA GAA TTC CCG GGG ATG TTG CGC AGC ACT T |
| P3247_AS_X | AAA AAA TCT AGA TTC CGG CTG CGC TCG GCG GAT T |
| P_SMa1635_E | AAA GAA TTC GAA GAG CCG CCA AGC GAG ATA T |
| P_SMa1635_X | AAA TCT AGA GGT AAT CCA TCC GGC ATT GAA A |
| P_SMa2245_E | AAA GAA TTC CGT CTT AGG TGA TTG GTT CAA GAA |
| P_SMa2245_X | AAA TCT AGA CCT TCA GTA GCG GCC AAG TGA A |
| ccrM_N | AAA AAA CAT ATG AAG TTC GGG CCG GAA ACC A |
| ccrM_X | AAA AAA TCT AGA ATC AGT TCA TCC CCG CCC GCA |
| ccrM_E | AAG AAT TCA GGA GGT AAA AAA ATG AAG TTC GGG CCG GAA ACC A |
| Sm_mucR_N | AAA AAA CAT ATG ACA GAG ACT TCG CTC GGT A |
| Sm_mucR_E | AAA AAA GAA TTC ACT TGC CGC GAC GCT TCC GA |
|  | **Oligonucleotides used for Real-time PCR (5’-3’)** |
| P169_F1 | GTA ATC CCA ACC TCA AAA AAT GAA |
| P169_R1 | GAG CGT TGC ACT TCT TAC GAT A |
| 1083_F1 | GAT CGG CCA GAT CTT GCG AA |
| 1083_R1 | CCC GCC CTG GCA TTA TCC AA |
| P1149_F1 | GAA GTA ATG GAT AGC GCG GAA |
| P1149_R1 | TAG ATC GAT GGT TCG TCG ATA TA |
| P2830_F1 | CCG ACC AAC TCG AAC GCG AA |
| P2830_R1 | GAA TAA ATA ACT AGT GGT GGG TAA |
| P2901_F1 | CGT TGC TTC GGC TTT GTG ACA A |
| P2901_R1 | CGA TGC ATA CAA TAC CGC CTA A |
| P3248_F1 | ACG ACA TAA TGT CGC ACC ACA A |
| P3248_R1 | ATG GCG GCG GTT GCG AGC AA |
| P1248_F2 | GCCATGGATGCCCCTGGATA |
| P1248_R1 | GCGACGAACTACGTCCTGAA |
| P3426_F1 | CGCCGGAAGACGAGGATTGAA |
| P3426_R1 | CCCTAACGCAGTTCGCGGAA |
| syn_F1 | CCA ATT GTG AGC GGA TAA CAA T |
| 290_R1 | CGT AAT CAT GGT CAT AGC TGT T |
| SMa1635_F1 | TAC AGT GCG CTC AAA AGC CAA TA |
| SMa1635_R1 | CGA GAC AAA TCT TTG CGT TCT GA |
| SMa2245_F1 | AAG CTC TAG CTC GGT AGA GAA |
| SMa2245_R1 | CAA AGT TTG GTC TTC ACA GGC AA |
| SMc01552_F1 | GCG AGC TTC AGT GCC GTG AA |
| SMc01552_R1 | CGC TTA TGT CGC TCA TGC GAA |
| PnagA_F1 | CCG TCC GCT CCG ATA ACA TA |
| PnagA_R1 | CCA AGC CCA ATG TGA TAC CAA |
| PpodJ_F1 | TTG GGG TCA ATC CCC TTA ACG CTC CAT |
| PpodJ_R1 | AAC CAG TGT TAA CCC TCT TGC TTT TGA A |
| PxylX_F1 | GCT GGT CAG ACA ACC TAC TT |
| PxylX_R1 | GGT CGC GGC TTT CCA ATC TT |
| parS_F1 | GAC ATC CCA AGA CTT GGA CAA |
| parS_R1 | TCA CGT GGA ACA TCC CCA ATA |

**Synthetic DNA** (from Integrated DNA Technologies, Coralville, Iowa, USA):

P*169** (5’-3’):

GACCCTGGTGTCGCCCGGCGACACGATCCGCATTGGTGAGCGCTTCTTCTAGTTGGCTTGCCAGCATTGTTCGCAAAGGCCCGGCGGTGACGCCGGGCCTTTTTCGTTTGCGCCGCGCACCGACGCGCTCACGATGAGGCTTTAGGCGAGCGCATCGATCGATCCGCCGCCTTCATAGCGCGTGTCTGGTTCGAGATGGCTCAGCTAATGCCTAGGGGCTTTG*T*CTCAAAAATATCAAGAAAAACAACGGCGAGGACGAGAATAAATATCCTTATACAGATTAAAATCTCGATGGGCGCACCCCGAGCTTCTTTATGCGGTTAGCGGGGATGTAATCCCAACCTCAAAAAATGAACTCCAATTCGAGTTCCAAGTCG*T*ATCTGTCAAAAAAGCTTCTCACTTTCGTGATAGAATATTTTCCCTCATAATATTCGGCCACGAATAGTG*T*ATCTAATTCGGCCCTGG*T*TTCCCGCAGATGGGG*T*ATCCTCTCTATCGTAAGAAGTGCAACGCTCAGGCGCCTTGTATTGAGCGCCGACTGTTCCGCAAAAACTGTCACAATTGCCGACTAAACACCCCCGGAGGCTTGATCGCCTGCGTTCGTCGCGGCTTGAGAAACCAGAGGGGCTCCTGTGAAGCTGTATAGAAACCTAATCCTCATGAGCT

[mutated G*T*NTC sites underlined]

P*2901**(5’-3’):

GAATTCAACCATTATAATTCGGGCACAAATCGATCGCTCGGCCGTACCGGTTTCGGGTTTGGGAAAGCGTTGCTCAGCGTCCGGAGGTCTCTGTGCGACAAATTGTCCGTTGCTTCGGCTTTGTGACAAATATCACAGAGTTGAAATGGTCGTG*T*ATCTTTCCAAATAGAATTGATAAATCCAAGGATATATTTCGGATAACTTTGTTACTGCGGCGTGCTTCTTTAGTAGATAGCGGAGATTTTAACGCG*T*TTCGCCGCTCTGTTATTGCTTTAGGCGGTATTGTATGCATCGAAATTCATCGTTTCGCGGTATGGTCGTACTTGAGTGCAGGTGCGTTATTGTTGACGTTAATGCTATGTTAGCCGTTTTGCTCGACCTCATTAACCTAATACGTTGCGGGCGCGGCGTGCGAGCGGGGGGCTCGGGGATATGTGGTTCAAAGAGATCGCCCGACGGCGTCGCGCCGAGACGCGCCGCCTGAAGGAACTGGAGAGCCTGGCCAAGGCCATGGAGCGGTCGCAGGCCGTGCTGGAACTGCGGCCCAATGCGACGGTCGTCCGCGCCAACGGCGCGTTCCAGCGCCTGACCGGCTACAAGCTT

[mutated G*T*NTC sites underlined]

*Thermoplasma acidophilum* methylase (*Ta1168*), codon optimized for *C. crescentus* (5’-3’):

CATATGCACCTGCGCCACATGTCGGGGCGTATGATCGGGGACCAGAAGACCGTGCTGAAGTTCGAAACGATGCTGAACAACGTGATCTCCGGCGACAGCATCGAGATCATGAAGCAAATCCCCGATAACAGCGTGGATCTGATCTTCGCCGACCCGCCCTACAACCTCCAGCTGGAAAACGAACTGTACCGCCCGAACGAAACGAAGGTCAACGGCGTGTCGGAGGACTGGGACAAGTTCCGCAGCTTCCAGGACTACGACGACTTCACCCTGAACTGGCTCAGCCAGTGCAAGCGCATCCTGAAGGAGTCGGGGACCATCTGGGTCATCGGGACCTACCACAACATCTTCCGCGTCGGCAAGATCATGCAAGATCTGGGCTTCTGGATCCTCAACGACATCGTGTGGATCAAGACGAACCCCATGCCCAACTTCAAGGGGACGCGCTTCAACAACGCCCATGAGACCCTCATCTGGGCCTCGAAGGACAAGGAATCGAAGTATACCTTCAATTACAAGACCATGAAGGCCTACAACGATGACCTGCAAATGCGCAGCGACTGGTACATCCCCATCTGTCAGGGTGACGAGCGCATCAAGATCAATGGCCAGAAGCTGCACCCGACCCAGAAGCCGGAAGCCCTGCTGTACCGCATCATCACCGCGACCAGCAAGCCGGGCGATATCGTCCTGGACCCCTTCGCCGGCACCGGCACCACCCTGGTGGTGGCCAAGAAGCTGGGTCGGTCGTTCATCGGCATCGAGAAGGAGCCGCTGTACGTCGACGCCTGCCGTGAGCGCCTGAAGAACACGGCCTCGTACCAGCAGAAGCTGCTCGACTACCCGCTGGAGATCCGGCCGAAGCGGGTCCCCTTTGGCAGCCTGATCGAGAACGGGTATGTGAAGGCGGGCGAATATCTGTACTCGCCGGACGGCGAGGCCCGCGCGCTGGTCCTGGCGAATGGCACCCTCTCGTACGAGGACAAGTACGGCTCGATCCACAAGATCAGCGCGATGATCCTGAATAAGCCGGCCAACAACGGCTGGGCGTTCTGGTACGTGAAGCGCGATGGCAAGCTGGTCAGCATCAACGACCTGCGCCAGAAGCTGCTGAAGGACCAGTACGCCAATCATCACGGCATCCAGTGAATTC

*Helicobacter pylori* methylase (*HPHPP1_1656*), codon optimized for *C. crescentus* (5’-3’):

CATATGGACTTCCTGAAGGAAAACCTGAACACCATCATCGAAGGCGACTGCCTGGAGAAGCTGAAGGACTTCCCGAATAAGTCGGTCGATTTCATCTTCGCGGACCCGCCGTACTTCATGCAAACCGAGGGCGAACTGAAGCGCTTCGAGGGCACCAAGTTCCAGGGCGTCGAAGACCACTGGGATAAGTTCGGCTCGTTCGAGGAGTATGACACCTTCTGCCTGGGCTGGCTGAAGGAGTGTCAGCGCATCCTGAAGGACAACGGCTCGATCTGCGTCATCGGCTCGTTCCAGAACATCTTCCGCATCGGTTTCCATCTCCAGAACCTCGGGTTCTGGATCCTCAACGACATCGTGTGGTACAAGAGCAATCCCGTCCCGAATTTCGCCGGCAAGCGCCTGTGTAATGCCCACGAAACCCTGATCTGGTGCGCGAAGCATAAGAACAACAAGGTGACCTTCAACTATAAGACGATGAAGTATCTGAACAACAACAAGCAGGAGAAGTCCGTGTGGCAGATCCCCATCTGCATGGGCAACGAGCGGCTGAAGGACGCGCAGGGCAAGAAGGTGCACTCGACCCAGAAGCCCGAGGCCCTGCTGAAGAAGATCATCCTGTCGGCGACCAAGCCGAAGGATATCATCCTGGACCCGTTCTTCGGCACCGGTACCACCGGGGCGGTGGCGAAGTCCATGAACCGGTATTTCATCGGCATCGAGAAGGACTCCTTCTACATCAAGGAGGCGGCCAAGCGCCTGAACTCCACCCGGGACAAGTCGGACTTCATCACCAACCTGGACCTGGAGACGAAGCCCCCCAAGATCCCCATGTCCCTGCTGATCAGCAAGCAGCTGCTGAAGATCGGCGACTTCCTGTACTCGTCGAACAAGGAAAAGATCTGCCAGGTGCTGGAGAACGGGCAGGTGCGCGACAATGAAAACTACGAAACGAGCATCCACAAGATGTCGGCCAAGTACCTGAATAAGACGAACCACAACGGCTGGAAGTTCTTCTATGCCTATTATCAGAACCAATTCCTGCTGCTGGATGAACTGCGGTATATCTGCCAGCGCGACTCGTGAATTC

*Haemophilus influenzae* methylase (HifGL_001636), codon optimized for *C. crescentus* (5’-3’):

CATATGATGAAGGAGAACATCAATGATTTCCTGAACACCATCCTCAAGGGGGATTGCATCGAGAAGCTGAAGACCATCCCCAATGAGTCGATCGACCTGATCTTCGCCGACCCCCCGTACTTTATGCAGACGGAGGGCAAGCTCCTGCGTACCAATGGCGACGAGTTCTCGGGCGTCGACGACGAGTGGGATAAGTTCAACGACTTCGTCGAGTATGACTCCTTCTGCGAGCTGTGGCTGAAGGAGTGCAAGCGCATCCTGAAGTCCACGGGCAGCATCTGGGTGATCGGCTCGTTCCAGAACATCTACCGCATCGGCTACATCATGCAAAATCTGGATTTCTGGATCCTGAACGATGTCATCTGGAATAAGACCAACCCCGTGCCGAACTTCGGCGGGACCCGGTTTTGCAATGCGCATGAGACGATGCTGTGGTGCTCCAAGTGCAAGAAGAACAAGTTCACCTTTAACTATAAGACCATGAAGCACCTCAACCAAGAAAAGCAGGAGCGCTCGGTCTGGAGCCTGAGCCTCTGTACCGGCAAGGAGCGCATCAAGGATGAAGAGGGCAAGAAGGCCCACTCGACGCAGAAGCCCGAGTCGCTGCTGTATAAGGTCATCCTGAGCTCCTCGAAGCCGAACGACGTGGTCCTGGACCCGTTCTTCGGTACCGGCACCACCGGCGCCGTGGCCAAGGCGCTGGGCCGGAACTACATCGGTATCGAGCGCGAACAGAAGTACATCGACGTCGCCGAAAAGCGGCTGCGCGAGATCAAGCCCAACCCGAACGACATCGAGCTGCTGAGCCTCGAGATCAAGCCGCCGAAGGTGCCCATGAAGACCCTGATCGAGGCCGATTTTCTGCGCGTCGGCCAGACCCTGTTCGACAAGAACGAAAACGCGATCTGCATCGTCACGCAGGACGGCAACGTGAAGGACAACGAGGAAACGCTGTCCATCCACAAGATGTCGGCGAAGTACCTGAACAAGACGAACAATAACGGCTGGGACTACTTTTATCTGTTCCGCAACAACAACTTCATCACGCTCGATTCGCTGCGCTACGAATATACCAACCAGTGAATTC

Attenuated *E. coli* phage T5 promoter followed by three GANTCs overlapping two CtrA-boxes:

GAATTCGGATCCAATTGTGAGCGGATAACAATTACGAGCTTCATGCACAGTGAAATCATGAAAAATTTATTTGCTTTGTGAGCGGATAACAATTGTAATATGTGGATTAAG**GACTC**CTTAAACCC**GAGTC**TTAAA**GACTC**GTTAAGGTCTAGA

[CtrA-boxes underlined, GANTC sites in bold]

**References**

1. Evinger M, Agabian N. Envelope-associated nucleoid from *Caulobacter crescentus* stalked and swarmer cells. J Bacteriol. 1977;132(1):294-301.

2. Fumeaux C, Radhakrishnan SK, Ardissone S, Theraulaz L, Frandi A, Martins D, et al. Cell cycle transition from S-phase to G1 in *Caulobacter* is mediated by ancestral virulence regulators. Nature communications. 2014;5:4081. doi: 10.1038/ncomms5081. PubMed PMID: 24939058; PubMed Central PMCID: PMC4083442.

3. Zweiger G, Marczynski G, Shapiro L. A *Caulobacter* DNA methyltransferase that functions only in the predivisional cell. J Mol Biol. 1994;235(2):472-85.

4. Wright R, Stephens C, Zweiger G, Shapiro L, Alley MR. *Caulobacter* Lon protease has a critical role in cell-cycle control of DNA methylation. Genes Dev. 1996;10(12):1532-42.

5. Gonin M, Quardokus EM, O'Donnol D, Maddock J, Brun YV. Regulation of stalk elongation by phosphate in *Caulobacter crescentus*. J Bacteriol. 2000;182(2):337-47.

6. Casse FB, C.; Julliot, J.S.; Michel, M.; Dénarié, J. Identification and characterization of large plasmids in *Rhizobium meliloti* using agarose del electrophoresis. Journal of General Microbiology. 1979;113:229-42.

7. Becker A, Ruberg S, Kuster H, Roxlau AA, Keller M, Ivashina T, et al. The 32-kilobase exp gene cluster of *Rhizobium meliloti* directing the biosynthesis of galactoglucan: genetic organization and properties of the encoded gene products. J Bacteriol. 1997;179(4):1375-84. Epub 1997/02/01. PubMed PMID: 9023225; PubMed Central PMCID: PMC178839.

8. Simon R, Priefer U, Puhler A. A broad host range mobilization system for in vivo genetic engineering: transposon mutagenesis in gram negative bacteria. Nat Biotechnol. 1983;1:784-90.

9. Gober JW, Shapiro L. A developmentally regulated *Caulobacter* flagellar promoter is activated by 3' enhancer and IHF binding elements. Mol Biol Cell. 1992;3(8):913-26.

10. Ardissone S, Fumeaux C, Berge M, Beaussart A, Theraulaz L, Radhakrishnan SK, et al. Cell cycle constraints on capsulation and bacteriophage susceptibility. eLife. 2014;3. doi: 10.7554/eLife.03587. PubMed PMID: 25421297; PubMed Central PMCID: PMC4241560.

11. Skerker JM, Shapiro L. Identification and cell cycle control of a novel pilus system in *Caulobacter crescentus*. Embo J. 2000;19(13):3223-34.

12. Khan SR, Gaines J, Roop RM, 2nd, Farrand SK. Broad-host-range expression vectors with tightly regulated promoters and their use to examine the influence of TraR and TraM expression on Ti plasmid quorum sensing. Appl Environ Microbiol. 2008;74(16):5053-62. doi: 10.1128/AEM.01098-08. PubMed PMID: 18606801; PubMed Central PMCID: PMC2519271.

13. Thanbichler M, Iniesta AA, Shapiro L. A comprehensive set of plasmids for vanillate- and xylose-inducible gene expression in *Caulobacter crescentus*. Nucleic Acids Research. 2007;35(20):e137. doi: 10.1093/nar/gkm818.
